# Conceptual priorities shape individual gaze patterns during naturalistic visual attention

**DOI:** 10.1073/pnas.2604369123

**Published:** 2026-06-12

**Authors:** Amanda J. Haskins, Katherine O. Packard, Caroline E. Robertson

**Affiliations:** ^a^ Department of Psychological and Brain Sciences, Dartmouth College, Hanover, NH 03755; ^b^ Department of Psychology, University of California, San Diego, CA 92093

**Keywords:** attention, computational modeling, cognitive neuroscience, virtual reality

## Abstract

Our visual landscape consists of not only people, places, and objects (e.g., “soldier,” “stadium,” “flag”) but also the conceptual relationships that unite them (e.g., “patriotism”). Because conceptual knowledge varies across individuals, this level of structure may support individualized patterns of attentional selection during naturalistic scene viewing. Here, we ask whether individuals’ gaze patterns reflect, in part, latent attentional priorities organized in conceptual space. Participants (N = 61) freely explored a diverse set of immersive real-world scenes (N = 100) in head-mounted VR while their gaze position was continuously recorded. We modeled gaze behavior using spatial, visual, and conceptual feature spaces, leveraging embeddings from large vision and language models, to uncover the latent priorities guiding individuals’ unique patterns of selective attention across environments. Individuals exhibited stable and idiosyncratic gaze patterns across scenes and test–retest sessions, consistent with trait-like individual differences in attention. Spatial, visual, and conceptual feature spaces each explained unique variance in individual gaze patterns, with conceptual features contributing variance beyond that explained by spatial and visual features alone. Notably, language-model–based predictions were particularly effective at capturing these individualized patterns. Together, these findings indicate that naturalistic visual attention is structured at multiple levels—including a conceptual level—revealing stable individual differences in how people sample and prioritize information across complex visual environments.

## Significance

Stepping into a new visual environment, we immediately start to explore that environment with our eyes. What factors shape how we selectively allocate our attention? Participants explored 360°, real-world environments while their gaze was tracked, and we modeled the hidden priorities guiding their attention. We found that gaze patterns across diverse scenes are individually specific and stable across time—akin to an “attentional fingerprint.” Importantly, large language models, which capture abstract conceptual relationships, predicted these patterns better than vision models, suggesting that conceptual knowledge plays a crucial role in individual selective attention. These results show that stable, idiosyncratic conceptual priorities shape how individuals encode their visual environments.

To understand our visual world, we take in the scene content around us one glance at a time. Along the way, we make choices about what information to sample and what to ignore—bringing our individual knowledge bases, expectations, and goals to bear on this process. In this sense, gaze provides a powerful window into cognition, revealing how the brain prioritizes information to make sense of complex visual environments.

Understanding the priority map that guides selective attention is a central goal of psychology. Traditionally, gaze has been modeled as the outcome of a dynamic competition between the relative priority of “bottom–up” (i.e., salience) and “top–down” (i.e., meaning) scene features for a viewer (1–3). Importantly, this traditional dichotomy offers little insight into how the brain computes priority with respect to the context in which a scene is viewed (4, 5). Selective attention is strongly shaped by extrinsic context: the experimental task- and reward-conditions under which a scene is viewed (6–9). Intuitively, selective attention is also shaped by intrinsic context: an individual’s unique knowledge bases, personality traits such as curiosity and creativity, and even genetic factors (10–16). Yet the contributions of intrinsic context to the attentional priority map remain largely unexplored. In particular, it is unknown whether conceptual-level knowledge acts as a latent factor shaping how individuals allocate attention during natural viewing.

This gap is critical given current theories of selective attention, which position the viewer as an active information-seeker whose internal models guide exploration of the visual world (17–20). As the viewer scans a complex scene (e.g., attending to a “hat,” “sticker,” or a “ball”) they draw on conceptual knowledge (e.g., knowing that “balls with stickers on them are often for sale”) to formulate and test hypotheses about how objects relate to each other and what they afford (e.g., “is the ball for sale?” vs. “is this ball softball team practice gear?”) (21). In this way, an individual’s conceptual knowledge—formed over time through experience with statistical regularities in the world—provides a backdrop for attentional selection during scene understanding. Yet, despite evidence for reliable object-level priorities among viewers (22–24), the latent structure of conceptual-level attentional priorities, and their contribution to individual differences in gaze behavior, remains largely unexplored.

Here, we address this gap by testing whether conceptual knowledge beyond the visual domain shapes individual patterns of real-world selective attention. We hypothesize that an individual’s gaze patterns during natural scene viewing reflect—in part—their stable, endogenous conceptual priorities—that is, systematic tendencies to prioritize certain kinds of meaning or relationships across diverse environments. To test this, we developed a framework that models gaze behavior in the conceptual feature space of a large language model (LLM). In brief, LLMs learn high-dimensional representations that capture the co-occurrence structure of concepts in natural language, providing an approximation of conceptual-level relationships that extend beyond visual similarity (25). Importantly, this feature space can distinguish between the context-dependent meaning of visually similar objects (e.g., a ball as “merchandise for sale” vs. “softball team practice gear”), going beyond the visual-level feature space of vision models which represent image statistics, but not context-dependent meaning (26, 27).

**Fig. 1.**
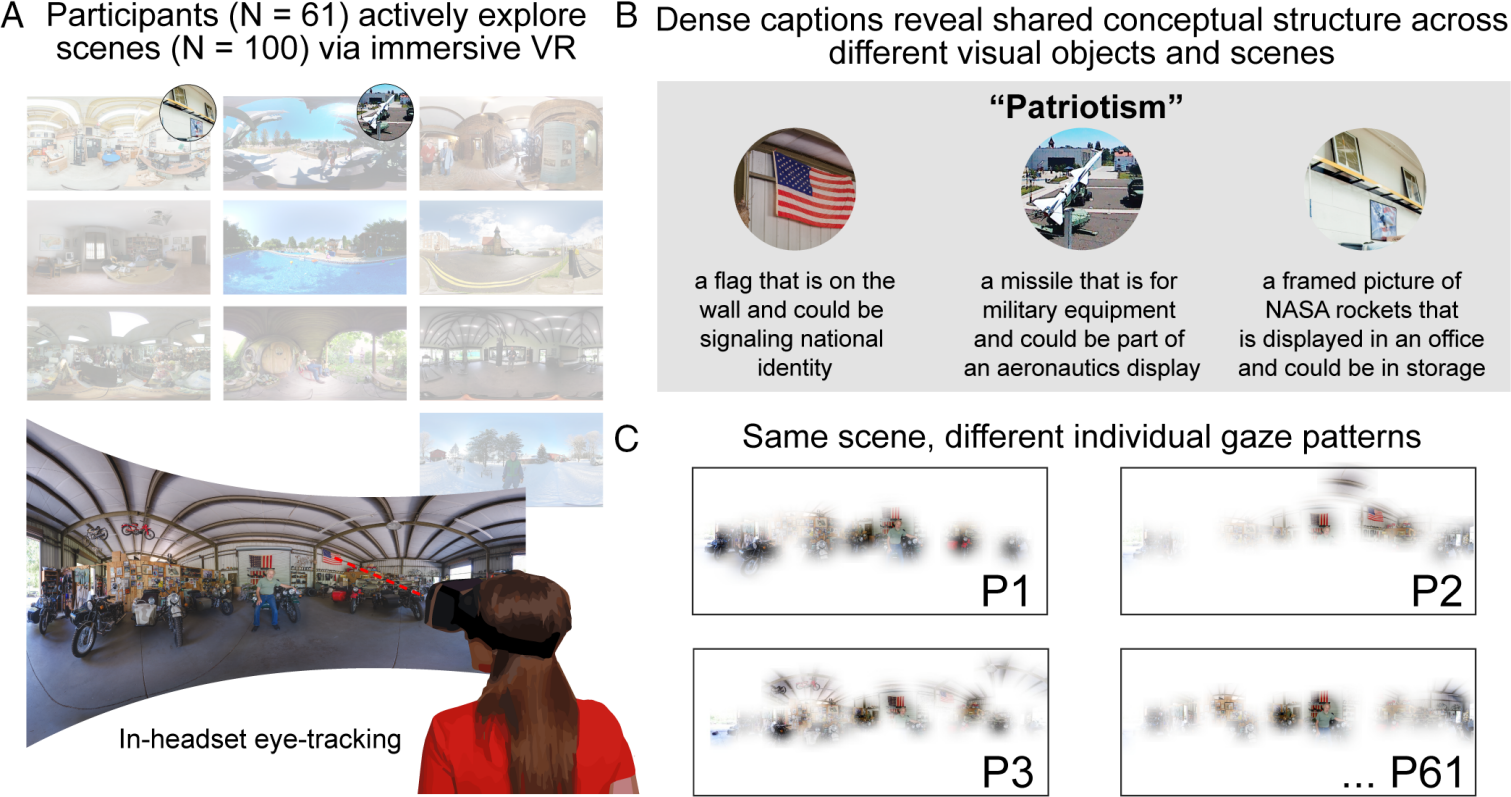
Paradigm and approach to modeling latent “conceptual” priorities in real-world gaze behavior. (*A*) Participants (N = 61) wore head-mounted VR displays with binocular eye-trackers and freely explored immersive, 360° real-world scenes (N = 100; 16 s per scene). Scenes depicted complex, real-world environments (e.g., an automobile shop, a public swimming pool, an airport) containing diverse objects, people, and settings so that individual participants could express different information-seeking priorities. (*B*) Scenes were densely captioned by online raters, enabling the identification of abstract conceptual themes (e.g., “patriotism”) that span different object types and contexts, such as flags, military equipment, and framed images, despite substantial visual dissimilarity. (*C*) Same scene, different individual gaze patterns. Smoothed, duration-weighted gaze maps are shown for multiple participants, highlighting reliable individual differences in gaze allocation (“gaze fingerprints”) despite identical visual input.

We combined conceptual features from an LLM with visual features from a state-of-the-art vision model and spatial information to model gaze behavior in a large cohort of participants (N = 61) who freely explored a diverse set of real-world environments (N = 100) using head-mounted virtual reality with in-headset eye tracking (Fig. 1*a*). With this setup, participants could actively explore scenes via head-, eye-, and body-movements, magnifying the opportunities for idiosyncratic viewing behavior while retaining experimental control over stimulus content (28). Using a stacked regression framework, we quantified the unique contribution of conceptual, visual, and spatial feature spaces to individual gaze patterns while controlling for shared variance between them.

Our results reveal that individual differences in naturalistic gaze behavior are reliable and structured at multiple levels, including a conceptual level that contributes uniquely beyond visual and spatial factors. Importantly, conceptual contributions are stable across scenes and time, indicating enduring individual differences in how people prioritize information during real-world scene understanding. Together, these results suggest that gaze behavior offers a window into how internal knowledge structures shape visual attention in everyday environments.

## Results

**Fig. 2.**
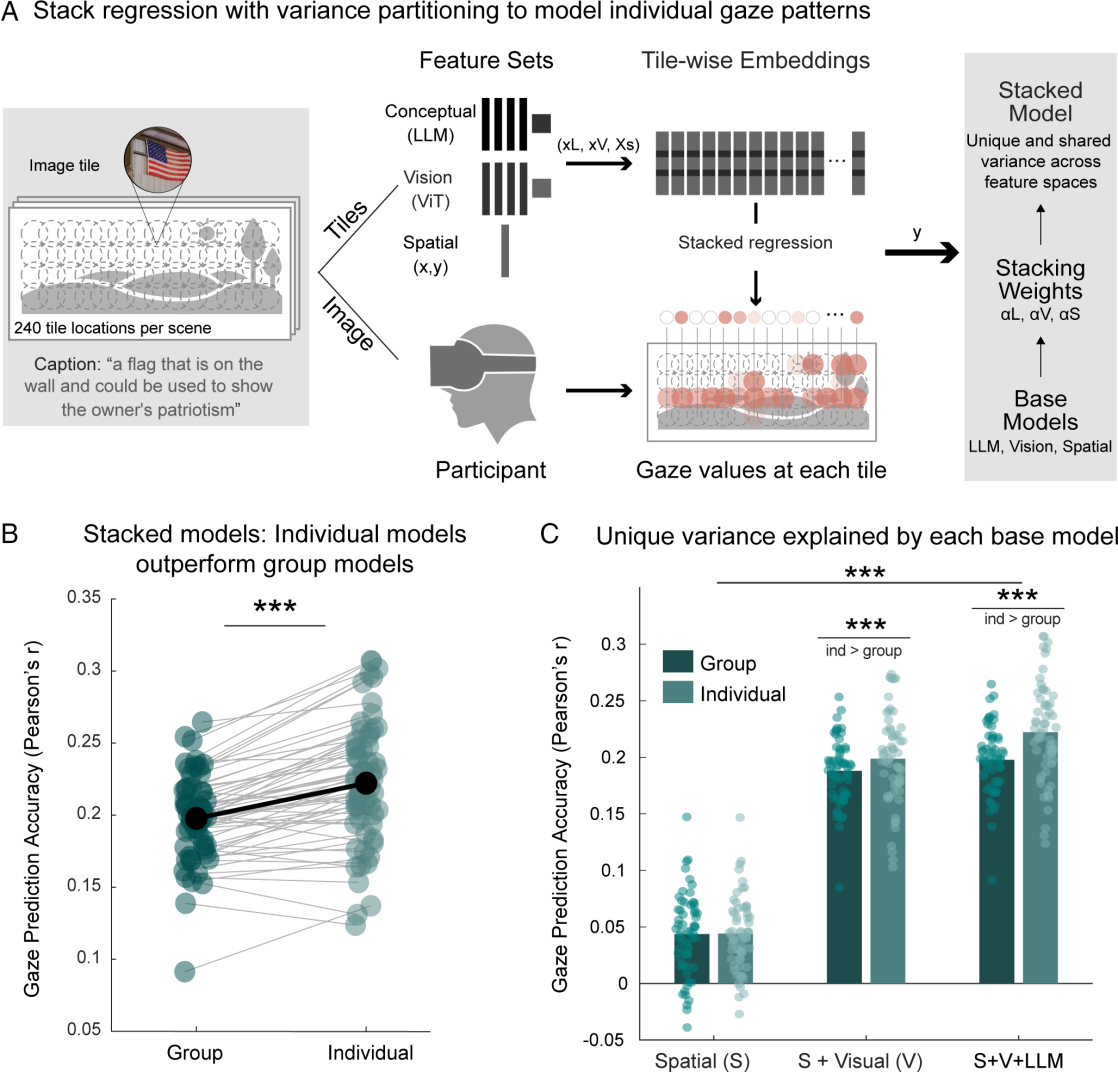
Stacked regression reveals individually specific and conceptually structured gaze patterns. (*A*) Stacked regression and variance-partitioning framework. Each immersive scene is divided into spatially defined image tiles. For each tile, features are extracted from multiple feature spaces: spatial coordinates (x, y), visual embeddings from a vision transformer (ViT), and conceptual embeddings from a large language model (LLM) derived from human-generated tile captions. For each participant, separate base gaze models are fit within each feature space to predict tile-wise gaze values. Predictions from these base models are then combined using a stacked regression that learns scene- and participant-specific stacking weights (α_L, α_V, α_S), enabling decomposition of gaze prediction performance into variance that is shared across feature spaces and variance that is uniquely attributable to each feature space. (*B*) Prediction accuracy for individual gaze behavior (Pearson’s r) are shown for stacked models trained at the group level (fit to gaze behavior averaged across participants) and at the individual level (fit separately for each participant). Individual stacked models significantly outperform group-level models, indicating that individualized modeling captures structure in gaze behavior that is not present in average group gaze maps. (*C*) Unique variance explained by each feature space. Model–data correlations show variance explained by spatial features alone (S), spatial plus visual features (S + V; isolating variance uniquely attributable to visual features beyond spatial), and spatial, visual, and conceptual features combined [S + V + LLM (i.e., stacked models); isolating variance uniquely attributable to conceptual features beyond spatial and visual]. Importantly, individual models show larger gains from adding visual and conceptual feature spaces relative to group models, with the largest gains from adding conceptual feature spaces, indicating that higher-level feature spaces capture participant-specific structure in naturalistic gaze behavior beyond shared spatial biases.

Our overarching hypothesis was that, beyond well-documented spatial and visual biases in attention (22, 24, 29), individuals’ gaze patterns during naturalistic scene viewing are shaped by endogenous conceptual priorities (Fig. 1). To test this, we used a stacked regression and variance-partitioning framework to model gaze behavior across a large and diverse set of real-world scenes (Fig. 2). In this framework, separate base models predict gaze from spatial coordinates, visual features, or conceptual embeddings, and a stacked model combines their out-of-sample predictions to assess whether each feature space explains unique variance beyond that shared with correlated predictors (e.g., conceptual information such as “shelter” often co-occurs with overhead spatial locations and roof-like visual features).

Using this framework, we first asked whether gaze behavior in an active, immersive context could be accurately modeled when spatial, visual, and conceptual information are considered together. We then asked whether conceptual (LLM-based) models explain unique variance beyond spatial and visual base models, and whether this variance reflects individually specific and stable aspects of gaze behavior across scenes and time. Importantly, Visual and language feature spaces were derived from transformer models with matched architecture (ViT and BERT), and all results held when feature spaces were matched either for dimensionality or for variance explained (*Methods* and *SI Appendix*, Fig. S2).

Throughout the Results, we distinguish between Group Gaze Models, which are trained on gaze behavior averaged across participants, and Individual Gaze Models, which are trained separately for each participant using their own gaze behavior. At each level, we fit three Base Gaze Models—a Spatial Base Model, a Visual Base Model (ViT), and a Conceptual Base Model (LLM)—as well as a Stacked Gaze Model that combines their predictions, to dissociate shared versus individualized components of visual attention.

### Gaze Models Capture Shared Structure in Group-Level Gaze Behavior.

Before turning to individual differences in gaze behavior, we first asked whether the stacked regression framework captures the shared structure of visual attention across viewers. Group-level gaze patterns in naturalistic scenes are known to be highly reliable, reflecting common spatial biases and content biases across participants (29–31). Establishing that our framework recovers this shared structure provides an important validation step and a baseline against which the predictions generated by Individual Gaze Models can be interpreted.

To model group-level gaze behavior, we averaged gaze distributions across participants for each scene and related these group gaze maps to tile-level feature representations using the stacked regression framework (Fig. 2*A*). For all analyses, gaze behavior was first represented as a duration-weighted map of fixation density for each scene. Using this map, the center coordinate within each scene tile was used to model and predict gaze (*Methods*). Specifically, we fit separate Spatial, Visual, and Conceptual base gaze models and combined their out-of-sample predictions using a stacked regression approach (i.e., the Stacked Model) (*Methods*). Model performance was evaluated using leave-one-scene-out cross-validation, such that the model was trained on N–1 scenes and used to predict the observed group gaze distribution on the held-out scene. Prediction accuracy was assessed by correlating the observed gaze distribution with the model prediction for each held-out scene.

This analysis revealed that each base model (i.e., Spatial, Visual, and Conceptual) explained significant variance in group-level gaze behavior, each yielding highly accurate group-level predictions across scenes (Spatial Base Model: *M* = 0.07, SD ± 0.16; Visual Base Model: M = 0.36, SD ± 0.18; LLM Base Model: M = 0.31, SD ± 0.14). Importantly, all three of these feature spaces also explained unique variance in gaze behavior, beyond their shared variance, as evidenced through comparisons between combined base models. In particular, spatial and visual base models together yielded highly accurate group-level predictions across scenes (Spatial + Visual: M = 0.36, SD ± 0.18), consistent with shared tendencies to attend to particular spatial locations and visual content in scenes. Yet, LLM features added unique variance beyond that of Spatial and Visual combined (All Features predictions: mean r = 0.38, SD ± 0.17; Spatial + Visual vs. All Features: t(59) = −6.25, *P* < 0.001, d = 0.81), indicating that higher-level semantic structure aligns with attentional patterns shared across viewers in group-level gaze data.

Together, these results show that the group-level stacked model successfully captures the reliable group-level structure of naturalistic gaze behavior and provides a principled baseline for examining whether these same feature spaces account for individualized patterns of visual attention.

### Gaze Models Accurately Predict Individual Gaze Patterns.

We next extended this framework to individual viewers, testing whether gaze models trained on an individual’s own data predict their gaze better than group-trained models —thereby distinguishing stable observer-specific structure in gaze behavior from noise around the group mean.

Both Group Stacked Models and Individual Stacked Models accurately predicted individual gaze behavior (Fig. 2*B*). Critically, Individual Stacked Models outperformed Group Stacked Models when predicting an individual’s gaze (Group Model vs. Individual Models: t(60) = −8.53, *P* < 0.001, d = 1.09), demonstrating that gaze behavior contains reliable observer-specific structure that generalizes across scenes. These results establish that gaze behavior at the level of individual observers is systematically predictable from spatial, visual, and conceptual scene features, setting the stage for dissecting feature-specific contributions.

### Visual and Conceptual Features Explain Unique Variance in Individual Gaze.

Next, we sought to understand feature-specific contributions to gaze prediction. To address this, we used a variance partitioning framework within a stacked regression model to examine whether spatial, visual, and conceptual feature spaces uniquely contribute when predicting individual gaze behavior with models that were either trained on Group-Level vs. Individual-Level data (Fig. 2*C*).

We started by considering Spatial Base models, incrementally adding visual and then conceptual features to assess their unique contributions. For Spatial Base Models, Group Gaze Models and Individual Gaze Models each predicted gaze (Group: M = 0.04, SD = 0.04; Individual M = 0.04, SD = 0.03). When visual features were added to the Spatial Base model, prediction accuracy increased, indicating that object-level visual information explains unique variance in gaze behavior beyond spatial biases. This improvement was evident for both group-level and individual-level models (Group: M = 0.19, SD = 0.03; S + V vs. S: t(60) = 26.96, *P* < 0.001, d = 3.45; Individual: M = 0.20, SD = 0.04; S + V vs. S: t(60) = 21.74, *P* < 0.001, d = 2.78).

Adding conceptual (LLM-derived) features to the spatial + visual model yielded a further reliable improvement in individual gaze prediction for both group-level and individual-level models (Group: M = 0.02, SD = 0.03 S + V + L vs. S + V: t(60) = 27.08, *P* < 0.001, d = 3.47; Individual: M = 0.22, SD = 0.04; S + V + L vs. S + V: t(60) = 23.85, *P* < 0.001, d = 3.05), indicating that conceptual representations explain unique variance in gaze behavior beyond that accounted for by spatial and visual structure alone. Together, these results demonstrate that individual gaze behavior reflects a combination of spatial biases, visual preferences, and higher-level conceptual structure.

### Conceptual Models Preferentially Capture Individual-Specific (vs. Shared Group) Variance.

While these analyses establish that both visual and conceptual features explain unique variance in gaze behavior, despite the correlations between these feature spaces, they do not indicate whether this unique variance primarily reflects structure that is shared across viewers or structure that differentiates individuals (Fig. 2*C*). Therefore, we next tested whether gaze models trained at the individual level outperform group-trained models across the different feature space combinations.

For spatial features, there was no benefit of individual-level training (S: Group vs. Individual, t(60) = −0.25, *P* = 0.8, d = 0.03), indicating that spatial biases primarily capture attentional structure that is shared across viewers. In contrast, individual-level models significantly outperformed group-level models for both combined feature spaces (S + V: Group vs. Individual, t(60) = −3.80, *P* < 0.001, d = 0.49; S + V + L: Group vs. Individual, t(60) = −8.53, *P* < 0.001, d = 1.09). This indicates that visual and conceptual feature spaces encode information that differentiates individuals beyond common viewing structure.

Critically, the magnitude of this individual-level benefit differed across feature space combinations. The individual-specific accuracy gain (Individual − Group) was significantly greater for S + V + L than for S + V combined models (t(60) = 13.35, *P* < 0.001, d = 1.71), indicating that conceptual features provide a substantially larger advantage in capturing individual differences in gaze behavior. A parallel analysis of base models (single feature spaces) revealed the same graded pattern: the benefit of individual-level modeling was greatest for conceptual features, intermediate for visual features, and minimal for spatial features (*SI Appendix*, Fig. S3).

Together, these results reveal a graded dissociation across feature spaces, with conceptual representations playing a disproportionate role in capturing individual-specific (vs. shared) attentional structure. In the next section, we ask whether conceptual feature spaces also account for what differentiates one individual’s gaze behavior from another’s.

### Conceptual Base Models Generate Individually Specific Predictions—Own Versus Other Model Comparisons.

Having shown that conceptual feature spaces explain unique variance in individual gaze behavior, we next asked whether this variance is individually diagnostic—that is, whether a given participant’s gaze behavior is better predicted by their own model than by models trained on other participants. This tests whether conceptual gaze models capture observer-specific structure beyond shared attentional biases.

For each participant, we compared the accuracy of gaze models trained on that participant’s data (“own”) to models trained on data from other participants (“other”). Using the same leave-one-scene-out procedure described above, we computed an “own-other difference score” for each participant—subtracting the average prediction accuracy for others’ models from the accuracy of the participant’s own model, with group-level predictions partialled out to isolate individualized variance.

**Fig. 3.**
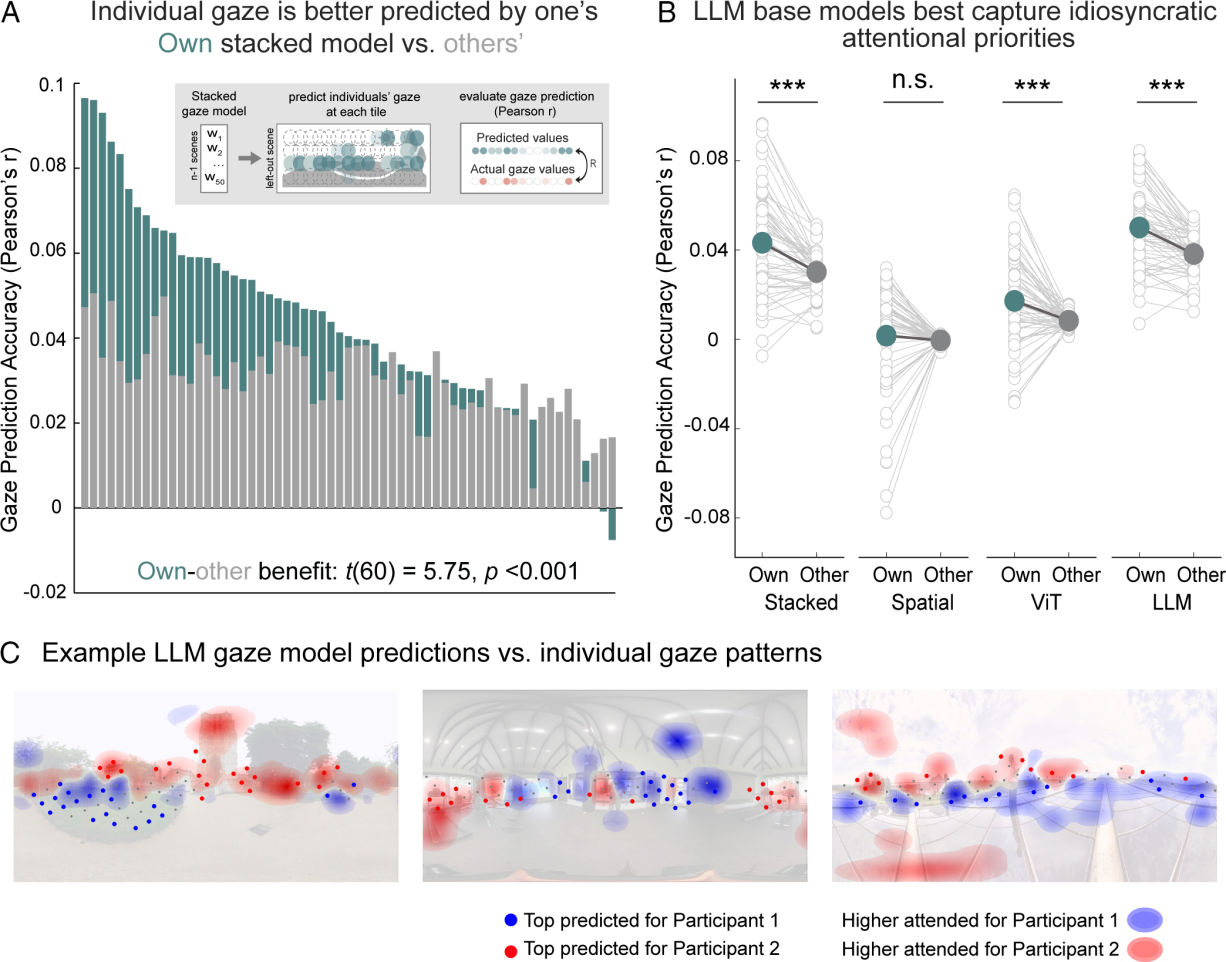
Conceptual feature spaces capture idiosyncratic attentional priorities in gaze behavior. (*A*) Own vs. other gaze prediction accuracy. For each participant (N = 61; bars sorted by own–other difference), gaze prediction accuracy is shown as the mean Pearson correlation (*r*) between predicted and observed tile-wise gaze values on held-out scenes. Teal bars indicate prediction accuracy from a participant’s own gaze model, trained on that participant’s gaze behavior in other scenes; gray bars show the mean accuracy obtained using models trained on other participants. Across participants, own models significantly outperformed other models (two-tailed paired *t* test). Insets schematize the modeling and evaluation framework. (*B*) Feature-space specificity of idiosyncratic gaze predictions. Paired points show own (colored) versus other (gray) prediction accuracy for stacked models and for base models derived from spatial, visual (ViT), or conceptual (LLM) feature spaces. Stacked and conceptual (LLM-based) models show robust own–other advantages, whereas spatial models do not, indicating that conceptual feature spaces preferentially preserve individually specific variance in gaze behavior (***P < 0.001; n.s., not significant). (*C*) Example scene-level predictions. Example photospheres illustrate predictions from LLM-based gaze models and observed gaze distributions for two participants viewing the same scenes. Despite shared spatial viewing biases, participants prioritize different scene regions, highlighting individually specific attentional patterns that generalize across environments.

The Stacked (Omni) Gaze Model showed a reliable own-other advantage (own: M = 0.04, SD = 0.02; other: M = 0.03, SD = 0.01; t(60) = 5.75, *P* < 0.001, d = 0.74), with nearly every participant better predicted by their own model than by others’ (Fig. 3*A*). This demonstrates that individual gaze patterns contain structured unique variation that distinguishes one individual from another, beyond shared group-level variance.

We next asked how spatial, visual, and conceptual priorities differentially contribute to this individuating effect. Own-other comparisons across base models revealed a dissociation that closely mirrored the individual-versus-group comparison (Fig. 3*B*). Conceptual (LLM-based) base models showed the strongest individuation: participants’ own conceptual models reliably outperformed models trained on other observers (own: M = 0.05, SD = 0.02; other: M = 0.04, SD = 0.01; t(60) = 7.23, *P* < 0.001, d = 0.93). In contrast, this own-other advantage was reduced for Visual (ViT) models (own: M = 0.02, SD = 0.02; other: M = 0.01, SD = 0.00; t(60) = 3.44, *P* < 0.001, d = 0.44) and statistically absent for spatial models (own: M = 0.00, SD = 0.02; other: M = 0.00, SD = 0.00; t(60) = 0.67, *P* = 0.25, d = 0.09).

Together, these results show that conceptual gaze models uniquely benefit from being tailored to individual observers, capturing aspects of visual attention that distinguish one person’s gaze behavior from another’s. In the final section, we ask whether these individually specific conceptual gaze patterns are stable across time and new visual environments.

### Conceptual Feature Contributions Emerge Over the Time Course of Scene Exploration.

**Fig. 4.**
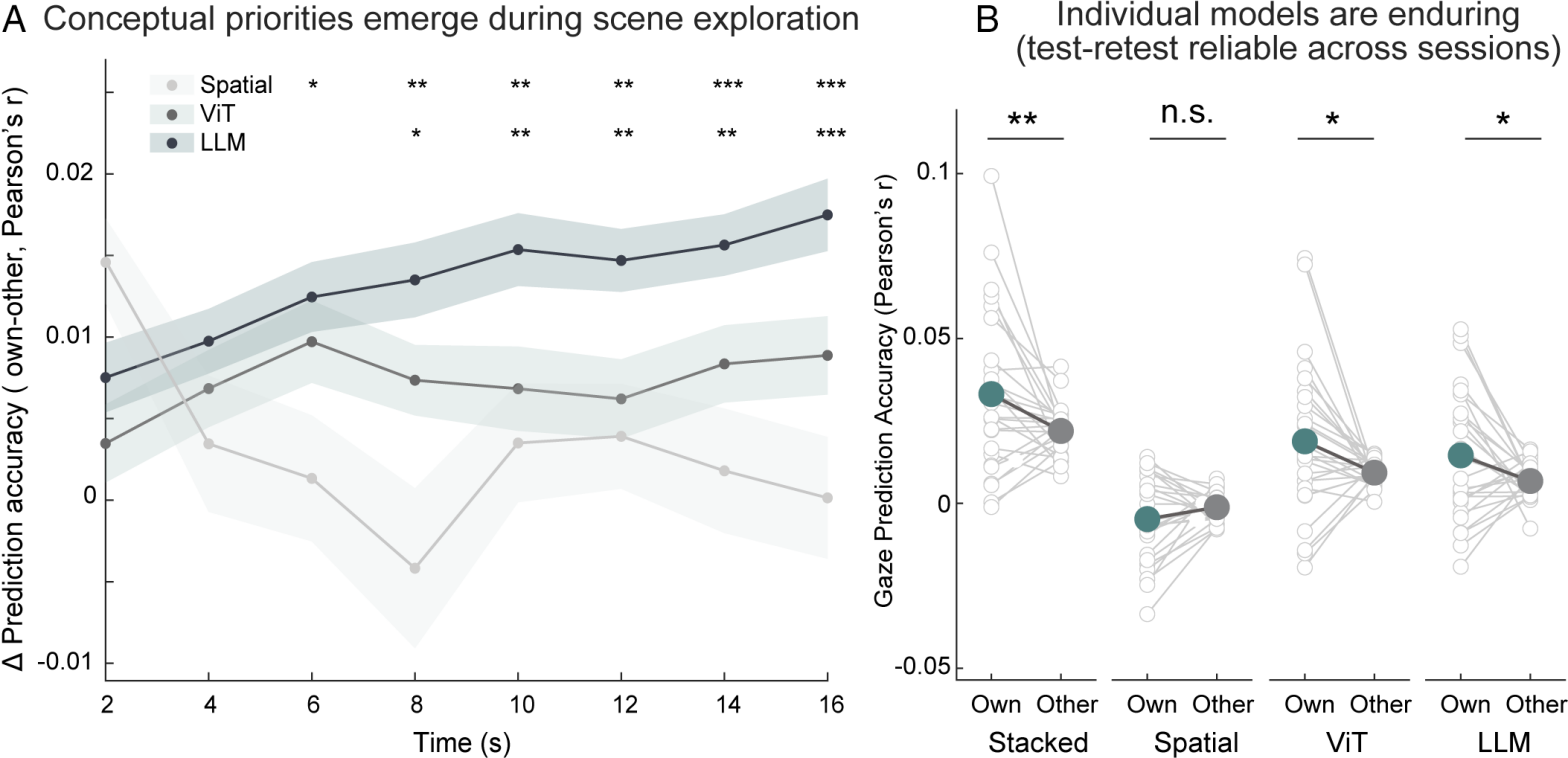
Conceptual feature spaces capture emerging and enduring individual differences in naturalistic gaze. (*A*) Time-resolved emergence of individual-specific attentional priorities during scene exploration. The y-axis shows the difference in gaze prediction accuracy between each participant’s own model and models trained on other participants (Δ = own − other). Curves show mean Δ accuracy over time for spatial (light gray), visual (ViT; teal), and conceptual (LLM; dark gray) base models; shaded regions indicate ±2 SEM. Stars mark time points where the LLM own–other benefit exceeds that of ViT (top) or spatial (bottom) models, indicating that conceptual priorities increasingly differentiate individuals during scene exploration. (*B*) Test–retest reliability of individual gaze models across sessions. Paired points show gaze prediction accuracy for each participant’s own model versus models trained on other participants (Δ own − other), evaluated on data from a second, retest testing session (Session 2). Results are shown for stacked (Omni), spatial, visual (ViT), and conceptual (LLM) models. Lines connect within-participant values; larger colored points indicate means. Individual stacked, visual, and conceptual models show reliable own–other advantages across sessions, whereas spatial models do not. For both plots: *P < 0.05, **P < 0.01, ***P < 0.001.

Whereas the analyses above ask which features explain gaze behavior as aggregated over the duration of scene viewing, we next ask when these features differentially contribute during scene exploration (Fig. 4*A*). Because conceptual embeddings capture information related to how objects in a scene relate to one another, we predicted that conceptual features would contribute relatively more at later stages of scene exploration, as scene understanding develops. To test this, we recomputed cumulative gaze maps and refit the stacked model at successive timepoints, quantifying partial correlations between predicted and observed gaze to track how spatial, visual, and conceptual contributions evolve over time.

This analysis revealed distinct temporal profiles across feature spaces (Fig. 4*A*). A repeated-measures ANOVA with factors of feature space (spatial, visual, conceptual) and time (2 s increments) revealed a significant main effect of feature space (Main Effect of Feature Space: *F*(2, 120) = 9.42, *P* < 0.001, η^2^p = 0.136) as well as a significant interaction between feature space and time (Feature Space × Time Interaction: *F*(14, 840) = 3.12, *P* < 0.001, η^2^p = 0.049).

Pairwise comparisons at each timepoint revealed that conceptual features significantly outperformed spatial features beginning at 6 s (t(60) = 2.31, *P* = 0.01, d = 0.30; FDR-corrected) and visual features beginning at 8 s (t(60) = 2.21, *P* = 0.02, d = 0.28; FDR-corrected; Fig. 4*A*). Spatial features were most predictive early in scene viewing, consistent with rapid, coarse spatial biases in initial sampling (e.g., equator biases). Visual feature contributions emerged shortly thereafter, potentially reflecting sensitivity to visually salient and object-level properties. Finally, conceptual (LLM-derived) features exhibited a more gradual increase in predictive power over time, ultimately exceeding spatial and visual predictors as viewing progressed.

Importantly, conceptual information was not absent early in viewing; rather the influence of conceptual feature spaces emerges progressively, increasing as observers accumulate information about scene structure and context. This temporal profile is consistent with our hypothesis that conceptual priorities increasingly shape attentional selection as scene understanding develops—possibly reflecting an internal model of how elements within a scene relate to one another. Together, these findings suggest that naturalistic gaze behavior reflects a dynamic integration of spatial, visual, and conceptual influences over time.

### Gaze Model Predictions for Each Feature Space are Stable Across Testing Session, Reflecting Enduring Attentional Priorities.

Finally, we asked whether these individually specific gaze patterns reflect enduring attentional priorities, rather than transient states (e.g., fatigue, mood) or measurement noise (Fig. 4*B*). To address this, we conducted a second, reliability testing session with approximately half of participants (N = 29, 26 after exclusions, *Methods*) roughly a week (7 to 9 d) after the first session. We reasoned that if gaze patterns are indeed a signature of an individual mind, and not a signature of a particular testing session, gaze models from Session 1 should be able to individuate participants in Session 2, across days and a new set of scenes (N = 40).

Consistent with this prediction, stacked models trained on each person’s own data (from Session 1) showed higher across-session prediction accuracy than models built from other participants [Own Stacked Model: M = 0.03 (SD = 0.02); Other Stacked Models: M = 0.02 (SD = 0.01)], yielding a significant across-session own–other difference (t(25) = 2.45, *P* = 0.01, d = 0.48; Fig. 4*B*).

Importantly, own-other difference scores for both LLM and ViT Base Models were reliable as well: LLM models (t(25) = 1.89, *P* = 0.04, d = 0.37), Visual models (t(25) = 2.06, *P* = 0.02, d = 0.40), but not Spatial models (t(25) = −1.43, *P* = 0.92, d = −0.28; Fig. 4*B*). Together, these results show that conceptual and visual gaze models capture stable, individually specific attentional structure that generalizes across days and novel visual environments, consistent with enduring attentional priorities.

### Conceptual Base Model Accuracy Improves with Increased Conceptual Information.

Having shown that conceptual (LLM-based) gaze models capture individually specific and stable aspects of visual attention, we next asked what information within the LLM feature space supports these predictions. Specifically, we tested whether LLM-based gaze prediction depends on access to higher-level conceptual information beyond object identity.

**Fig. 5.**
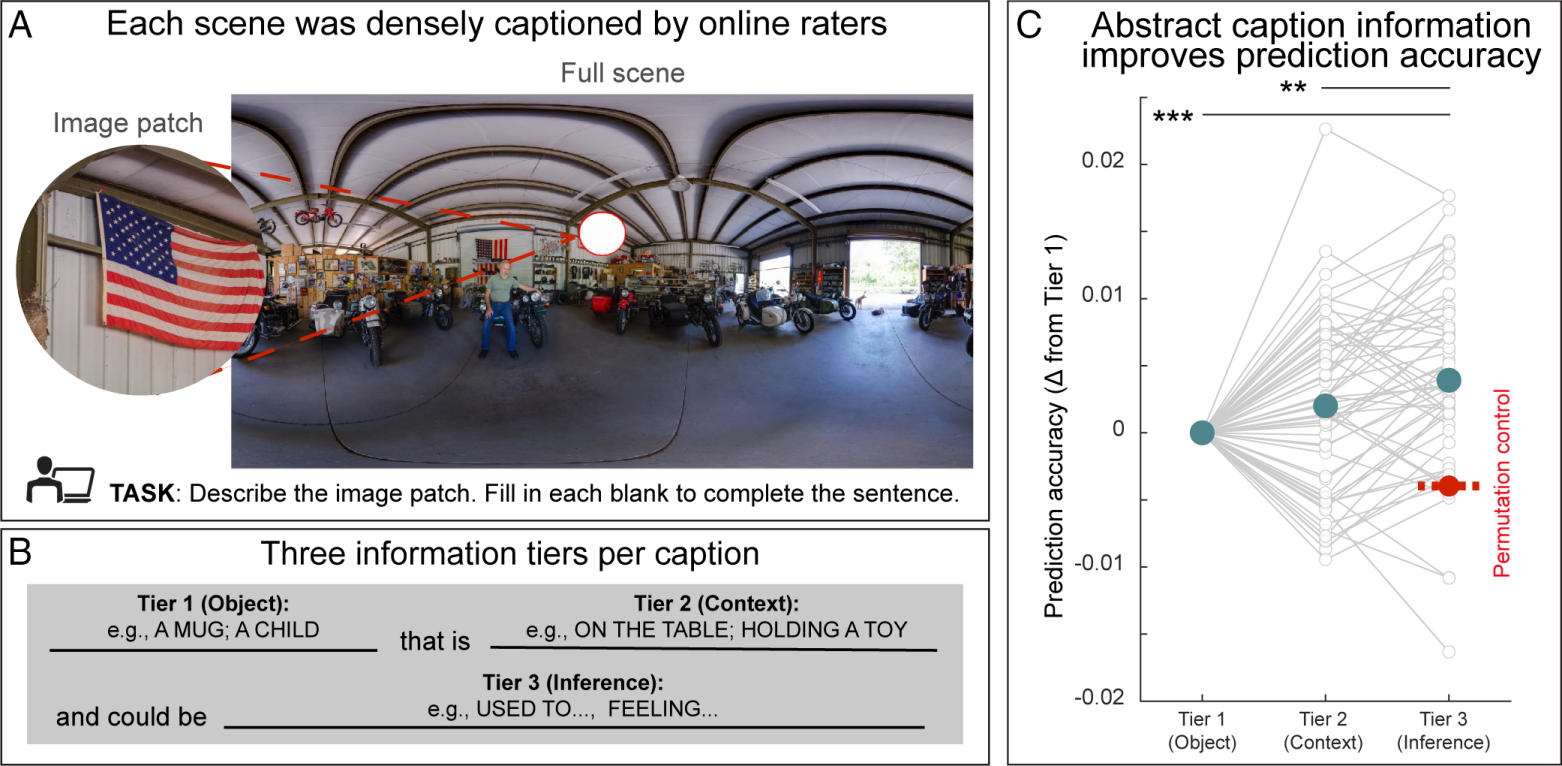
LLM-based gaze prediction improves with increasing conceptual depth of caption information. (*A*) Dense, structured captioning of scene patches. Each immersive scene was divided into spatially defined image patches, which were independently described by online raters using a standardized sentence-completion task. This procedure generated captions that incorporated object identity, relational, and inferential descriptions tied to each scene location (Tiers 1 to 3). (*B*) Three conceptual information tiers per caption. Captions were truncated to create three levels of conceptual information: Tier 1 (object identity; e.g., “a hat”), Tier 2 (relational context; e.g., “a hat that is on her head”), and Tier 3 (inferential or affordance-based information; e.g., “a hat that is on her head and could be keeping the sun from her eyes”). These tiers allowed conceptual depth to be varied while holding visual input constant. (*C*) Prediction accuracy scales with conceptual depth. Gaze prediction accuracy (Δ from Tier 1; Pearson’s r) is shown for stacked models incorporating Spatial and Visual (ViT) features together with LLM-derived features from captions truncated at each tier. Points show individual participants; lines connect within-participant values; larger colored points indicate means. Prediction accuracy increases with conceptual depth, with Tier 3 (inferential) captions yielding the largest gains. The red dotted line indicates the permutation control, in which Tier 3 caption content was randomly reassigned across scene locations, preserving linguistic richness while disrupting conceptual–visual alignment. Permuted captions do not outperform Tier 1, indicating that performance gains at Tier 3 cannot be explained by caption length or syntactic complexity alone, but specifically by inferential conceptual content. *P < 0.05, **P < 0.01, ***P < 0.001.

If individuation relies on conceptual structure in the LLM feature space, prediction accuracy should scale with the conceptual depth of the information used to represent each scene location. We tested this by exploiting the standardized structure of the tile captions provided by online raters, truncating them at three levels (Fig. 5 *A* and *B*): Tier 1 (object identity; e.g., “a hat”), Tier 2 (relational description; e.g., “a hat that is on her head”), and Tier 3 (inferential/affordance-based information; e.g., “a hat that is on her head and could be keeping the sun from her eyes”). This manipulation allowed us to isolate the contribution of increasingly abstract conceptual information to prediction accuracy, while holding visual input constant.

We constructed a Stacked Gaze Model that included Spatial and Visual (ViT) features alongside three Conceptual (LLM) feature spaces derived from captions truncated at each tier and evaluated prediction accuracy at each tier level (Fig. 5*C*). Prediction accuracy increased with conceptual depth: Tier 3 models outperformed Tier 1 (Tier 3: M = 0.190, SD = 0.03; Tier 1: M = 0.188, SD = 0.03; t(60) = 4.34, *P* < 0.001, d = 0.56, CI: [0.002, 0.006]) and Tier 2 models (Tier 2: M = 0.19, SD = 0.03; Tier 3 vs. Tier 2: t(60) = 2.75, *P* = 0.008, d = 0.35, CI: [0.001, 0.003]), while Tier 2 did not reliably outperform Tier 1 (t(60) = 2.42, *P* = 0.02, d = 0.31, CI: [0.000, 0.004]). This indicates that the advantage of Tier 3 information cannot simply be attributed to longer, more syntactically complex captions, but rather, to inferential conceptual content.

Importantly, all three tiers showed significant own-other advantages, demonstrating that individual-specific gaze patterns are captured even at the level of basic object identity (Tier 1: t(60) = 3.89, *P* < 0.001, d = 0.50), with increasing individuation as conceptual richness increased (Tier 2: t(60) = 5.09, *P* < 0.001, d = 0.65; Tier 3: t(60) = 4.78, *P* < 0.001, d = 0.61). These results remained qualitatively similar when partialling out group-level gaze predictions from each tier.

Finally, to rule out the possibility that LLM performance benefits simply from exposure to rich language rather than meaningful conceptual–visual alignment, we conducted a permutation control in which Tier 3 information (e.g., “and could be keeping the sun from her eyes”) was randomly reassigned across tile captions (*Methods*). This preserved linguistic richness while disrupting correspondence between conceptual content and visual context. Prediction accuracy dropped substantially under this manipulation (permuted Tier 3 vs. Tier 1: t(60) = −4.40, *P* < 0.001), falling below performance observed for Tier 1 captions alone (Fig. 5*C*).

Together, these results demonstrate that the advantage conferred by Tier 3 captions depends on meaningful alignment between inferential conceptual content and the corresponding scene location. This finding further supports the interpretation that Conceptual Base Model (LLM) captures conceptual priorities grounded in context-dependent meaning, rather than benefiting from rich language alone.

### Visualizing Individual Gaze Patterns Provides Human-Interpretable Insights Into Latent Conceptual Priorities.

The results above establish that Conceptual Base Models (LLM) capture unique, stable, and individually specific variance in visual attention. Yet, they do not indicate which visual content is conceptually linked for a given individual or what “concepts” may constitute a person’s priority map. Addressing this question is relevant for understanding whether Conceptual Base Model (LLM)s might capture conceptual (rather than purely visual) similarities across scene locations, as well as for gaining insight into different individuals’ idiosyncratic attentional priorities.

**Fig. 6.**
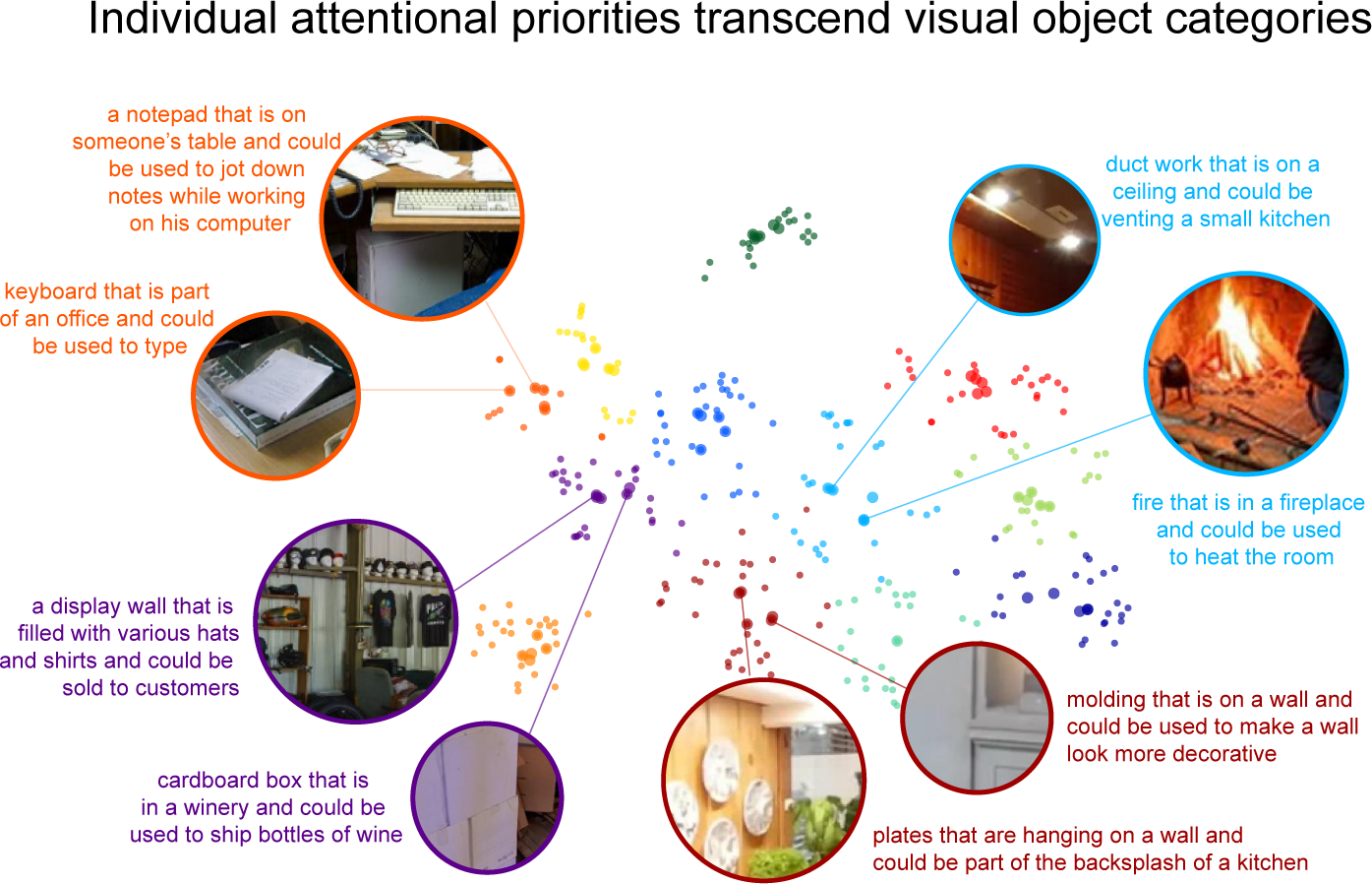
Conceptual priority maps reflect idiosyncratic attentional priorities that transcend visual object categories. Two-dimensional visualization of scene tiles uniquely prioritized by a single participant, derived from naturalistic viewing of 100 real-world scenes. Tiles shown correspond to the top 95th percentile of locations most uniquely attended by this participant relative to others and are embedded based on similarity in their LLM-derived conceptual representations (t-SNE visualization; clusters defined using k-means; see *Methods*). Insets show representative tiles and captions from selected clusters. Points cluster by shared conceptual themes rather than visual appearance, spatial location, or scene membership. For example, one cluster (orange) reflects a tendency to prioritize writing-related content (e.g., keyboards and notepads across scenes), whereas another reflects priorities related to interior decoration (e.g., molding and backsplashes).

To explore this structure qualitatively, we visualized individual participants’ high-priority scene locations using t-distributed stochastic neighbor embedding (t-SNE; see *Methods*; Fig. 6). For each participant, we first identified the subset of tiles that were most uniquely attended by that individual relative to others. Each such tile was then represented by its LLM-derived conceptual embedding, and t-SNE was used to project these high-dimensional embeddings into a two-dimensional space that preserves local similarity relationships. Importantly, the t-SNE projection reflects similarity in the conceptual feature space used by the gaze model, rather than similarity in visual appearance or spatial location.

These visualizations provide an intuitive depiction of how an individual’s attentional priorities cluster in conceptual space. In the example shown in Fig. 6, tiles that originate from different scenes and depict visually dissimilar objects nonetheless cluster together when they share abstract conceptual themes (e.g., writing-related items such as keyboards and notepads; interior design elements such as molding and backsplashes). These clusters offer a qualitative illustration of a participant’s “conceptual priority map,” suggesting that attentional priorities may be organized around abstract, context-dependent concepts that cut across scenes and object categories.

We emphasize that this analysis is intended as a visualization and hypothesis-generating tool rather than a statistical test. While this approach provides qualitative insights into the concepts a participant prioritizes—suggesting that high-priority tiles cluster around shared conceptual themes (e.g., writing; decoration)—further empirical testing is needed to validate any specific interpretations.

## Discussion

Our results show that gaze patterns offer insight into the conceptual priorities different individuals bring to bear while exploring their visual environment. Across a diverse set of real-world environments, people reliably differ in how they allocate attention, and these differences are structured at a conceptual level that extends beyond shared spatial biases and visual features. Notably, these conceptual gaze models are robust in two key ways: 1) they reliably predict gaze patterns across different testing days, indicating that they capture stable, trait-like attentional priorities; 2) and they generalize across diverse scenes, indicating that they are robust to everyday settings, object categories, and visual features. Overall, each individual leaves an “attentional fingerprint” on their environment—a signature of how conceptual knowledge, visual, and spatial biases collectively shape the sampling of visual information during everyday perception.

Previous research has identified reliable individual differences in gaze behavior, beginning with oculomotor biases of *how* different individuals move their eyes (e.g., fixation durations, saccade amplitudes) (32–34) and extending to stable individual preferences for visual object features and categories (e.g., faces or text) (22, 23, 35, 36). Our results build on this work by showing that idiosyncratic gaze patterns are structured not only by such biases but also by higher-level conceptual priorities that encompass visually dissimilar objects and scenes. Rather than reflecting preferences for specific object categories alone, individual gaze behavior can reflect conceptual themes that recur across environments (e.g., “patriotism”). For instance, an individual might direct attention to “patriotism” across vastly different environments (e.g., classrooms vs. airplane fields) and objects (e.g., flags vs. jets). In this way, individual gaze patterns reflect, in part, the distinctive conceptual priorities different individuals bring to bear when exploring their visual environment.

This conclusion is supported by two key lines of evidence. First, we find that conceptual (LLM-derived) features explain reliable variance in individual gaze behavior beyond that accounted for by spatial biases and visual feature representations, despite strong correlations among these feature spaces in natural scenes. Moreover, conceptual feature spaces provide a disproportionately large advantage for capturing individual-specific structure, as shown by larger individual-versus-group gains and stronger own–other individuation effects relative to spatial and visual models (ViT). Thus, although ViT represents what objects are in a visual scene, as well as how they visually co-occur in natural experience (due to its transformer architecture), it lacks the ability to represent how two different objects might be related by virtue of a more abstract concept (e.g., linking a flag and a jet via “patriotism”). Second, the Conceptual Base Model (LLM) accuracy increases with the availability of inferential or affordance-based information in scene captions, and drops when captions are restricted to object identity alone. Together, these results indicate that conceptual-level information plays a critical role in modeling individual attentional priorities beyond visual similarity or object-category structure. This account aligns with theories that emphasize proactive, meaning-based attentional selection in natural scenes (17–20, 31, 37, 38).

Our results dovetail with recent neuroimaging studies showing that the semantic structure of language embeddings can offer insight into the organizing principles of visual processing in the brain. LLM sentence embeddings have shown a surprising capability to predict fMRI activity in the visual cortex during scene viewing (39–41), and recover category-level organization of the cortex (40). Relatedly, the statistical structure of object co-occurrence in text and photographs has been shown to predict neural responses in object-selective and place-selective brain areas, respectively (42). Together with the present study, these findings show that contextual knowledge, as captured in the geometry of language model embeddings, shapes visual processing. Here, we extend this perspective by showing that such conceptual structure is expressed not only in neural representations but also in visual behavior, shaping the attentional priorities that guide how we sample the visual world through eye movements.

Why does a large language model, which has never received visual input, succeed at revealing individuating signatures in visual behavior? It is important to emphasize that the LLM feature space is built from *textual* training data—specifically training on next-word prediction in sentences from a large corpus of text—and thus is in no way grounded in visual experience. Accordingly, the Conceptual Base Model (LLM) has no access to the pixel-level visual content of scene regions underlying any given fixation. Instead, it encodes how linguistic descriptions of visual content tend to relate to one another in human language, reflecting patterns of co-occurrence and association across contexts.

Crucially, the geometry of LLM word embeddings has been shown to reflect rich dimensions of world knowledge (25), and to predict human judgments of the similarity of captioned visual scenes (43). Here, we argue that this statistical structure of natural language can be leveraged as a proxy for conceptual organization, allowing us to reveal latent conceptual priorities that shape selective attention during naturalistic scene viewing. Intuitively, it makes sense that we use world knowledge, gained through experience with statistical regularities in the sensory world and language itself (44), to scaffold selective visual attention, particularly in complex, real-world scenes where meaning must be inferred rather than read directly from visual input. Under these conditions, conceptual knowledge provides a scaffold for efficient, hypothesis-driven sampling of visual information, allowing observers to prioritize what is likely to matter from the earliest moments of scene exploration (45). This perspective emphasizes that individual differences in conceptual knowledge proactively shape how visual information is sampled as scene understanding unfolds.

Importantly, the conceptual attentional priorities captured here should not be interpreted as direct measures of personal preferences, beliefs, or values. Rather, they reflect stable, individualized patterns in how visual information is sampled during scene exploration, which may be shaped by a range of factors including experience, expectations, and contextual interpretation. Future work could test whether groups of individuals with different beliefs or prior experiences exhibit systematic differences in how conceptual-level information is prioritized during naturalistic visual exploration.

Immersive VR offers a unique balance between ecological validity and experimental control that is ideal for modeling attentional priorities across environments. It is well-understood that traditional, computer-based eye-tracking paradigms are not natural reflections of ecological attention: They use stimuli that are framed by a photographer to intentionally bias how a viewer’s visual attention is guided (29), which are presented on computer screens to stationary participants (28). Meanwhile, mobile eye-tracking offers more ecological validity but sacrifices stimulus display standardization and stimulus variety across participants. In contrast, immersive VR supports active, self-directed scene exploration, while maintaining large, diverse, standardized stimulus sets—affording the densely sampled naturalistic gaze behavior required to uncover stable individual differences in gaze patterns.

Several open questions remain. First, our analyses show that the relative contribution of conceptual information increases over the course of scene exploration (Fig. 4*a*), consistent with the idea conceptual priorities increasingly shape attention as scene understanding unfolds. However, these analyses do not yet capture the fine-grained temporal structure of gaze behavior—such as sequence-level transition dynamics—which may offer additional insight into how viewers switch between conceptual priorities over time (46, 47). Second, although our findings offer a human-interpretable “snapshot” of the conceptual features underlying individual gaze patterns (Fig. 5), future experimental work is needed to distill these features into namable priorities and generalizable axes that might vary across individuals, cultures, and clinical populations. Third, we focused on BERT as a specific LLM and ViT (which is matched to BERT for both dimensionality and architecture), as a representative vision model, but future work should benchmark gaze prediction performance across many deep neural network models and architectures (48) to further explore the latent structure of individual attentional priorities. Moreover, it is likely that multimodal (vision and language) models, like CLIP (49), would outperform an LLM alone, by leveraging both visual and linguistic feature spaces—particularly given our finding that visual and conceptual feature spaces each explain unique variance in gaze behavior.

Finally, the idea of stable, individual-specific attentional signatures—“attentional fingerprints”—carries implications for cognitive, clinical, and computer science. First, if different individuals sample unique visual information, how do we converge on common understandings of our environment? Although most variance in eye movements is shared across observers, differences in attentional priorities may have cascading consequences for later memory and cognition. Second, these results have clinical relevance. A large body of work has documented differences in selective visual attention in clinical conditions, including autism, where gaze behavior may serve as an early non-verbal diagnostic marker. Our results suggest that reduced attention to key semantic categories, like faces, in autism could be the “tip of the iceberg,” reflecting broader differences in conceptual-level priorities. Future work should test whether attentional differences in autism observed in naturalistic viewing paradigms (50–53) are best accounted for by visual or conceptual-level models. Third, our findings raise important privacy considerations for emerging technologies. Although gaze patterns in our study are not uniquely identifying in a biometric sense, they are individual-specific within our dataset, reflecting stable differences in what people attend to across scenes and time. It remains an open question whether, as datasets and modeling approaches scale, such individual specificity could enable increasingly fine-grained individuation or support inferences about individuals’ cognitive or conceptual profiles. These possibilities underscore the need for careful consideration of how gaze data are collected, stored, and used in VR/AR systems that incorporate eye-tracking.

All in all, our findings show that active visual exploration is guided by individual information-seeking priorities that transcend visual categories into abstract conceptual spaces. By leveraging the feature space of a large language model, we introduce a principled framework for modeling gaze behavior and uncovering the latent conceptual organization that shapes how people sample their visual environment. We hope that this method provides a broadly applicable framework for studying the conceptual priorities that underlie selective attention in different populations and contexts. Taken together, our results suggest that gaze behavior carries a stable, trait-like signature—an attentional fingerprint that reflects how each individual’s conceptual knowledge, experiences, and expectations guide perception in the real world.

## Methods

61 participants freely explored immersive 360° real-world scenes (16 s/trial) presented in head-mounted virtual reality while their eye movements were continuously recorded using in-headset eye-tracking. Each scene was densely captioned by online human raters to generate natural-language captions capturing object identity, relational context, and inferred affordances. We modeled individual gaze distributions using stacked regression models that predicted fixation density from three feature spaces: spatial location, visual features derived from a deep vision model, and conceptual features derived from a large language model applied to tile captions. Model performance and unique contributions of each feature space were assessed using cross-scene and cross-session generalization and variance partitioning. Full details of experimental procedures, data preprocessing, modeling, and statistical analyses are provided in the *SI Appendix*, *SI Methods*.

## Supplementary Material

Appendix 01 (PDF)

## Data Availability

All code and data have been deposited in Code Ocean (https://doi.org/10.24433/CO.7277423.v1) (54).
