# Supplementary material for: Conceptual priorities shape individual gaze patterns during naturalistic visual attention
Source: Proc Natl Acad Sci U S A. 2026 Jun 12;123(24):e2604369123. doi: 10.1073/pnas.2604369123 (PMC13273318; doi:10.1073/pnas.2604369123)
Supplement: Supplementary file 1 — Appendix 01 (PDF) [file pnas.2604369123.sapp.pdf]

Supplementary Information

Supplementary Methods

Participants

66 adults (*n* = 36 female; mean age 20.67 +/- 3.42 STD years) participated in this study (Session 1; Supplementary Table 1,2). A subset of these participants (*n* = 29) returned for a second testing session (Session 2) approximately one week later (between 7-9 days). For each participant, Sessions 1 and 2 were scheduled at approximately the same time of day (i.e., morning or afternoon). All participants reported either normal or corrected-to-normal vision, no colorblindness, and no history of seizures or other neurological conditions. Written consent was obtained from all participants in accordance with the Declaration of Helsinki and with a protocol approved by the Dartmouth College Institutional Review Board before the study. Following participant exclusion (criteria detailed below), data were analyzed from 61 participants in Session 1 and 26 participants in Session 2 (Supplementary Table 2).

Materials

Stimuli

A diverse set of 100 immersive, real-world photospheres were used in this study. Photospheres were sourced from online photo sharing websites and were curated to contain a balance of indoor versus outdoor environments (Supplementary Fig.1). Each photosphere depicted a complex, everyday environment (e.g., a cafe, home, park) rich in semantic content (e.g., people, objects, settings) and featured one to three people. To ensure that each photosphere was informationally rich, such that individual participants could express different information-seeking priorities, we specifically selected photospheres in which a set of pilot participants reliably attended to both social (i.e., people) and nonsocial information sources.

Stimulus presentation via headmounted virtual reality

The paradigm was programmed using custom scripts written in C# using Unity (unity3d.com) and presented to participants via headmounted virtual reality display (Oculus Rift, Development Kit 2; low persistence OLED screen; 960 x 1080 resolution per eye; ~100° field of view; 75 Hz refresh rate). Participants stood wearing the headset throughout the testing session, affording the opportunity to naturally explore each photosphere by moving their eyes, head, and body.

Eyetracking technical specs

Binocular, in-headset eye-trackers monitored gaze location (Pupil Labs: 120 Hz sampling frequency, 5.7 ms camera latency, 3.0 ms processing latency; 0.6 visual degrees accuracy, 0.08 visual degrees precision). Gaze coordinates were recorded with custom scripts written in C# for Unity.

Procedure

Testing Sessions

At the start of each testing session (Session 1 and Session 2), participants performed a 21-point eyetracking calibration routine, which was repeated after every 10 trials or whenever calibration drift was detected. Before of each trial, a pre-trial fixation task, which required the participant to fixate a target for 5 seconds before proceeding to the next trial, ensured both the calibration's accuracy and that the initial fixation on each trial was at screen center for all participants.

On each 16s trial, participants were instructed to naturally explore a real-world photosphere via eye, head, and body movements: "look around each scene naturally, like you would look around a new place in your daily life." In Session 1 (N=60 trials), each trial displayed a novel photosphere from the stimulus set of 100 photospheres, with the same 60 scenes displayed in a randomized order across participants. In Session 2 (N=60 trials), participants viewed 40 novel photospheres, as well as 20 repeat photospheres that had previously been showed in Session 1. After each trial, participants were permitted to take a break as needed before advancing to the next pre-trial fixation screen. Participants were offered a break to remove the headset after completing 30 study trials, after which they would return to the calibration routine.

Eyetracking data pre-processing, participant exclusion, and gaze map generation

Eyetracking samples were excluded if: 1) pupil detection confidence was low (below 0.5), 2) no data were collected (e.g., a blink occurred), or 3) the logged gaze location fell outside the field of view of the headmounted display. In addition, trials were excluded if: 1) confidence was below 0.5 for more than 35% of the trial, 2) pre-trial fixation deviation exceeded 5° visual angle (DVA), or 3) the participant scanned less than 60 percent of the photosphere (yaw). Participants were excluded entirely if fewer than half of trials remained following trial-level exclusion (Session 1: n = 5; Session 2: n = 3; see Supplemental Table 1 for eye-tracking data quality metrics).

Gaze data were processed using a custom toolbox developed to analyze in-headset eyetracking data in immersive virtual reality environments (28, 54). Raw eye-

tracking coordinates (*x, y*) and head position (*pitch, yaw, roll*) were first rectified to compute a continuous stream of gaze positions in the immersive photosphere. Next, fixations were defined as periods in which the mean absolute deviation of gaze position was less than 50°/s (55), with the corresponding fixation position (x,y) defined as the centroid of gaze samples within each fixation. Fixations shorter than 100 ms, as well as the first fixation in each trial (which was biased by the mandatory pre-trial fixation at screen center), were excluded from further analysis.

For each trial, fixation density maps were generated by plotting duration-weighted fixations in equirectangular coordinates. To account for distortions inherent in equirectangular projections, fixation density maps were smoothed using separable 1D Gaussian filtering with latitude-dependent filter widths (56). Specifically, horizontal smoothing was performed with a Gaussian window width that varied inversely with the cosine of latitude (pitch angle), calculated as baseWidth × (1/cos(pitch)), where baseWidth was set to 200 pixels. This inverse-cosine scaling results in narrower smoothing at the equator and progressively wider smoothing toward the poles, compensating for the increasing pixel density per degree of visual angle at higher latitudes. Filtering was applied bidirectionally (forward and reverse) along each row, with results summed to avoid edge artifacts at the longitudinal seam. Vertical smoothing was performed with a fixed Gaussian window width of 200 pixels, applied bidirectionally along each column. Smoothed fixation maps were then normalized on a scale from 0.1 to 1 to prevent extreme fixation durations from dominating correlations while preserving relative spatial structure. These final "gaze maps" were used for all subsequent analyses. Note that, because gaze maps were derived from duration-weighted fixation density maps, regions of high gaze density reflect both fixation frequency and dwell time, providing a continuous measure of attentional allocation rather than discrete fixation counts.

Constructing Conceptual Feature Spaces for Real-World Photospheres (MTurk and BERT)

To characterize the conceptual information in each real-world photosphere, we developed an approach that combined the rich conceptual knowledge of human raters with the high-dimensional feature space of a large language model (LLM). Each photosphere was first decomposed into a set of overlapping image tiles (N = 300), evenly sampling the scene (tile diameter: 18 visual degrees). Next, we obtained sentence captions for each image tile from human raters (N > 2500; 5 raters / image tile) on Amazon Mechanical Turk ("AMT"). Each task (AMT "HIT") contained 10 trials. On each AMT trial, one image tile was presented side-by-side with an equirectangular projection of the full photosphere, and an AMT participant was asked to provide a sentence caption of the content of each tile with reference to the broader context of the photosphere. To structure this sentence caption, raters were given a fill-in-the-blank captioning task with three separate responses

(“This is a ____ that is ____ and could be ____”). This captioning prompt was designed to elicit rich, contextualized captions that captured three tiers of information: Tier 1) the identity of any object depicted in that tile (e.g., “a hat”); Tier 2) relational information in the broader scene context (“that is on her head”) and Tier 3) inferential/affordance information relating to what that object might be doing in the broader scene narrative (“and could be keeping the sun from her eyes”). By standardizing the grammatical structure of each sentence caption, we ensure that the primary source of variation across image tiles is in semantic and conceptual content, rather than syntactic form, which BERT is somewhat sensitive to (57). Each tile in a HIT was sampled from a unique photosphere in the stimulus set, and raters were prevented from providing more than one caption per tile; thus, each HIT was completed by five independent raters.

Captions were subsequently filtered to eliminate low quality or otherwise unusable captions. Specifically, we excluded any captions that utilized the task example language (e.g., “a crowd of people”) or contained words from non-English languages from the analysis. Captions included in the analysis were preprocessed to remove spelling errors and redundant phrases (e.g., participant retyped “and could be” in one of their responses). Because viewers rarely make fixations at extreme latitudes (28, 58), we did not obtain sentence captions for the 20% of tiles located at the photosphere poles (i.e., the top and bottom 10%).

We then transformed each tile caption into a sentence level embedding using a large language model, *BERT* (Bidirectional Encoder Representations from *Transformers)* (59). Outlier embeddings (i.e., a caption embedding that deviated substantially from the other four caption embeddings on the same tile) were identified using a hierarchical clustering algorithm (60) and excluded from the analyses (less than 0.5% of captions). For each tile, we then averaged the remaining valid embeddings (from up to five raters) to produce a mean embedding for subsequent analysis. Thus, the visual information at each tile location was transformed into a feature vector (embedding) in the representational space of a large language model (BERT).

Characterizing non-conceptual feature spaces (visual and spatial)

As a control model to the LLM, we used a vision model, the visual transformer model (ViT; (61)) trained on image classification, to model the visual content depicted at each image tile. Importantly, these two models (BERT and ViT) have analogous transformer architectures and matched dimensionality (N = 768 dimensions). For each image tile described above, we also obtained a ViT embedding by extracting the top level [CLS] token embedding, representing the entire image tile. All in all, this approach allowed us to represent the visual content at each tile location as a feature vector in the high-

dimensional feature space of either a large language model (BERT) or a vision model (ViT).

As a second control model to the LLM, we used a spatial model to address the possibility that individually specific gaze patterns reflect systematic motor differences (e.g., tendency to look upward) that coincide with spatial regularities in conceptual information, rather than conceptual priorities themselves. For example, ceilings and tarps share both conceptual-level relatedness (i.e., they both provide shelter) and spatial coordinates (i.e., they are both located in upper portions of photospheres). Thus, we characterized the spatial coordinates of each tile in two dimensions (X,Y coordinates on the equirectangular photosphere projection). Spatial gaze models for each participant were then generated using these tile coordinates in the same manner as the language or vision model features.

Dimensionality reduction for high-dimensional feature spaces (language & vision)

We identified the primary sources of variation within the photosphere tiles in our stimulus set by applying principal components analysis (62) to the LLM and ViT embeddings for all photosphere tiles. This dimensionality reduction mitigates the "curse of dimensionality" (original N=768) and was performed before each key analysis. For each feature space, we selected the number of principal components (PCs) required to capture approximately 80% of the variance across tile embeddings. This procedure yielded 50 PCs for the LLM embeddings and 225 PCs for the ViT embeddings. We applied this dimensionality-reduced representation consistently across all analyses reported. To ensure that differences in the number of PCs (i.e., 50 PCs vs. 225 PCs) between feature spaces did not drive our results, we confirmed that all key findings remained qualitatively similar when using matched dimensionality (50 PCs for both LLM and ViT feature spaces; see Supplementary Fig. 2).

Analyses

Analyses overview

We used stacked regression to predict gaze distributions from spatial, visual, and conceptual feature spaces using leave-one-scene-out cross-validation, with prediction accuracy quantified as the tilewise Pearson correlation between predicted and observed gaze values. We used structured variance partitioning to quantify each feature space's unique contribution beyond variance shared with the others (Lin et al., 2024), operationalized as the improvement in prediction accuracy when a feature space was added to the others (e.g., All Features − Spatial + Visual). Individuation was quantified using own–other partial correlations controlling for group-level predictions, and reliability

was assessed across scenes and across sessions by testing generalization from Session 1 to Session 2.

Throughout the manuscript, Group Gaze Models refer to models trained on group-averaged gaze data, whereas Individual Gaze Models refer to models trained separately on each participant's own gaze data to capture observer-specific attentional structure.

Building stacked gaze models of participants' gaze patterns

For each participant, we built a stacked encoding model that combined predictions from three feature spaces: Spatial (tile coordinates in equirectangular space), Visual (ViT embeddings), and Conceptual (LLM embeddings). This stacked regression framework, adapted from stacked encoding approaches developed for combining feature spaces in fMRI (63), enables estimating the relative contribution of correlated feature spaces to gaze prediction while accounting for variance shared across predictors. The stacked modeling procedure consisted of two levels. At the first level, we trained separate ridge regression models for each feature space independently (i.e., base models). Then, at the second level, we learned optimal weights to combine predictions from these base models. All analyses used a leave-one-scene-out cross-validation scheme: for each scene, models were trained on the remaining scenes and evaluated on the held-out scene.

First-level base models. Methods followed from Lin et al., 2024. For each feature space *(Spatial, Visual, and Conceptual)*, we used ridge regression to predict a participant's gaze values (duration-weighted attention at each tile location) from the feature representations of those tiles. L2 regularization was used to prevent overfitting, with the regularization parameter (λ) selected independently for each feature space via 5-fold cross-validation, ensuring unbiased evaluation of each lambda value on data not used for training. We tested λ values between 10^{-6} and 10^{9} and selected the value that minimized prediction error on held-out folds. This cross-validation procedure also generated estimates of each model's performance on the training scenes, which were used to compute the stacking weights described below.

Second-level stacking weights. Predictions from the three first-level models were combined using a weighted linear combination with weights alpha = [alpha_Spatial, alpha_ViT, alpha_LLM]. For each held-out scene (and each participant), stacking weights were estimated by solving a constrained least-squares quadratic optimization problem that minimizes out-of-sample prediction error on the training scenes, following the stacked generalization framework (63). The weights were constrained to be non-negative and sum to 1, such that they reflect the relative importance of each feature space. Under this formulation, weights reflect the contribution of each base model to reducing generalization

error in the presence of correlated predictors, rather than independent explanatory importance.

***Final predictions*.** After computing the stacking weights using separate cross-validation folds, each first-level ridge regression model was retrained on all training scenes using the selected regularization parameter. Weighted predictions were then generated for the held-out scene. This process was repeated for each scene in a leave-one-scene-out manner, yielding cross-validated predictions for all scenes. Critically, because the stacking weights were learned separately for each held-out scene, stacking weights were scene-specific for each participant.

Evaluating prediction accuracy. Prediction accuracy was quantified as the Pearson correlation between predicted and observed gaze values across tile locations within each scene. For each scene, gaze behavior was summarized as a duration-weighted attentional distribution over image tiles, with each tile represented by a single value corresponding to the smoothed gaze density evaluated at the tile center (rather than pixel-wise comparisons across the full image). Predicted gaze maps were constructed in the same tile-based space. Accuracy was computed for individual base models (e.g., LLM predictions multiplied by the LLM stacking weight α_LLM), combined feature subsets (e.g., Spatial predictions multiplied by α_Spatial + Visual predictions multiplied by α_Visual), and the full stacked model. For each participant, accuracy values were averaged across valid scenes.

Evaluating the unique variance accounted for by LLM features

Throughout the manuscript, we operationalize the unique contribution of a feature space as the improvement in gaze prediction accuracy when that feature space is added to a model containing the other feature spaces, equivalent to variance partitioning in the stacked regression framework.

To test whether conceptual (LLM) features contribute uniquely to gaze prediction beyond spatial and visual features, we compared stacked models that included versus excluded the LLM feature space. Stacked models were fit at both the group level (using group-averaged gaze data) and the individual level (using each participant's gaze data), following the leave-one-scene-out stacked regression procedure described above.

Prediction accuracy was computed by correlating observed gaze values with model predictions for two feature-space combinations: (1) Spatial + Visual features (sum of weighted Spatial and Visual base model predictions) and (2) All features (sum of all three weighted predictions). We used paired t-tests to compare prediction accuracy between these two model combinations.

Evaluating the reliability of individual gaze models

To assess the reliability of individual gaze patterns, we compared the prediction accuracy of individual-level stacked models (trained on each participant's own gaze data) to the group-level stacked model (trained on group-averaged gaze data) when predicting each individual's gaze on held-out scenes. We used a paired t-test to compare prediction accuracy across participants. Superior performance of individual models would indicate reliable observer-specific structure.

Evaluating individual-level training benefits across feature spaces

To test whether certain feature spaces preferentially capture individual-specific versus shared gaze structure, we compared models trained on individual-level versus group-averaged gaze data. This comparison was performed separately for each base model (Spatial, Visual, and Conceptual) and for combined base models (e.g., Spatial + Visual vs. All Features).

For each feature space or feature-set combination, we evaluated how well group-trained and individual-trained models predicted each participant's gaze on held-out scenes. Model predictions were weighted using stacking weights from the corresponding full stacked model (group-level weights for group-trained models; individual-level weights for individual-trained models).

We computed the individual-level training benefit as the difference in prediction accuracy between individual-trained and group-trained models. To compare benefits across feature spaces, we tested pairwise differences of (Individual – Group) benefits between feature spaces (e.g., LLM – ViT). We used paired t-tests to compare group-trained versus individual-trained models within each feature space and feature space combination, and we compared the magnitude of this benefit across feature spaces to determine whether conceptual features (LLM base models) showed larger individual-level training benefits.

Evaluating individual specificity through own-other comparisons

We next tested whether gaze patterns are specific to each individual. To test this, we tested whether each participant's gaze patterns were uniquely captured by their own model relative to models from other individual participants. This analysis goes beyond the individual-versus-group comparison, evaluating whether individual gaze patterns are not only distinct from the group average but are uniquely specific to each individual.

For each participant and each scene, we computed the partial correlation between that participant's observed gaze and the predictions from every participant's stacked model (including their own), controlling for the group-level model predictions. This partialing

isolates individual-specific variance beyond what is captured by shared group-level gaze patterns.

We then computed "own" prediction accuracy as the partial correlation between observed gaze on a held-out scene and predictions from the participant's own model, averaged across scenes, and "other" prediction accuracy as the partial correlation with predictions from other participants' models, averaged across participants and scenes. To test for statistical significance, we used a paired t-test (right-tailed) to compare own versus average other prediction accuracy across participants. This analysis was performed for each base model (Spatial, Visual, and Conceptual) as well as the full stacked model.

Evaluating temporal dynamics of feature space contributions

To examine whether the contributions of spatial, visual, and conceptual feature spaces to individual-specific gaze behavior differ over the timecourse of scene exploration, we recomputed gaze model predictions as a function of cumulative viewing time.

For each time window (2, 4, 6, 8, 10, 12, 14, and 16 seconds following scene onset), we computed cumulative gaze distributions by aggregating fixations from scene onset up to that timepoint. We then rebuilt the entire stacked regression model (including re-learning stacking weights) using these cumulative gaze distributions, following the same leave-one-scene-out cross-validation procedure described above. This generated time-specific stacked models for each participant at each time window.

For each time window and feature space (Spatial, Visual, and Conceptual), we computed own-other difference scores following the same procedure described above: partial correlations controlling for group predictions, with own-other difference calculated as own prediction accuracy minus average other prediction accuracy.

To test whether feature spaces differed in their temporal profiles, we conducted a repeated-measures ANOVA with factors of Feature Space (Spatial, Visual, Conceptual) and Time (8 timepoints), using own-other difference scores as the dependent variable. To determine when conceptual features significantly exceeded spatial and visual features, we conducted pairwise comparisons (one-tailed t-tests: LLM > Spatial, LLM > ViT) at each timepoint, correcting for multiple comparisons using the Benjamini-Hochberg false discovery rate (FDR) procedure.

Evaluating cross-session stability of gaze predictions

For the subset of participants who completed a second testing session, we trained stacked models on Session 1 gaze data and used these models to predict Session 2 gaze behavior, following the same stacked regression procedure described above. PCA

loadings derived from Session 1 features were applied to Session 2 features to ensure consistent representations across sessions.

Cross-session stability was assessed using own–other comparisons, computed as partial correlations controlling for group-level predictions. Analyses were performed for each base feature space (Spatial, Visual, Conceptual) and for the full stacked model. Statistical significance was evaluated using paired-samples, one-tailed t-tests comparing own versus other prediction accuracy across participants.

Evaluating the role of conceptual abstraction in gaze prediction

To examine whether the predictive power of conceptual (LLM) features depends on access to higher-level conceptual information beyond object identity, we leveraged the hierarchical structure of the tile captions collected from human raters. Captions were generated in a consistent, incremental format that allowed us to systematically manipulate the level of conceptual information by truncating captions at three levels: Tier 1 (object identity only; e.g., "woman"), Tier 2 (object identity plus relational/descriptive details; e.g., "woman who is wearing a hat"), and Tier 3 (full captions including inferential or affordance-based information; e.g., "woman who is wearing a hat and could be keeping the sun from her eyes").

We next constructed a stacked gaze model with five feature spaces: Spatial, Visual (ViT), and three Conceptual feature spaces. The three Conceptual feature spaces were created by computing LLM embeddings for: (1) Tier 1 truncated captions containing only object labels, (2) Tier 2 truncated captions containing object labels plus relational information, and (3) Tier 3 full captions containing all three levels of information. Each set of embeddings was dimensionality-reduced via PCA (50 components per tier), and the stacked model was trained following the same leave-one-scene-out cross-validation procedure described above.

We evaluated prediction accuracy for each tier's weighted predictions (e.g., T1 × alpha_T1) by correlating them with observed gaze. We compared prediction accuracy across tiers using paired-samples t-tests, testing whether progressively richer conceptual information improved gaze prediction.

Permutation control. To test whether improved performance with higher-tier features depends on meaningful alignment between conceptual content and visual context (rather than simply richer language), we conducted a permutation control analysis. We randomly reassigned either Tier 2 or Tier 3 caption content across tile locations within each scene, creating permuted caption sets: Tier 1 + permuted Tier 2, and Tier 1 + Tier 2 + permuted Tier 3 (e.g., “a hat that is on her head / and could be part of an aeronautics display”). This

manipulation preserved complexity while disrupting conceptual-visual correspondence. LLM embeddings were recomputed from these permuted captions, and entire 5-feature stacked models (including the permuted tier) were rebuilt following the same procedure. We compared prediction accuracy of these permuted models to the intact Tier 1 model using paired-samples t-tests to determine whether disrupting conceptual-visual alignment eliminated the performance benefit for higher tiers.

Visualizing example participants' conceptual-level gaze models

In Fig. 6, to visualize the conceptual-level priorities that structure a participant's gaze patterns, we used t-distributed stochastic neighbor embedding (t-SNE), a non-linear method for mapping high-dimensional data in two-dimensional space.

First, we identified uniquely high priority tiles for each participant through a two-stage filtering process. For each of the 60 scenes shown during Session 1, we identified tiles where a participant's gaze density exceeded the 95th percentile within that scene. We then computed a uniqueness score for each tile by calculating how many participants selected it as a high-priority tile (top 95th percentile) across all scenes. This count was z-score normalized and inverted, such that tiles selected by fewer participants received higher uniqueness scores. We retained only tiles with uniqueness scores above 0.7 (corresponding to the 25th percentile of the across-participant distribution), indicating regions that were distinctively prioritized by individual participants rather than universally attended across the sample.

We applied t-SNE dimensionality reduction to the LLM embeddings of each participant's top tiles, followed by k-means clustering (k=12) on the resulting 2D embedding space (64). For each cluster, we identified the 5 most central tiles (nearest neighbors to the cluster centroid in t-SNE space) and visualized their associated text labels to interpret cluster meaning. Example tile captions were drawn randomly from the set of captions (N = 5) provided by raters for each cluster's centroid tiles.

General statistical methods. All analyses were performed across participants using paired-samples t-tests or repeated-measures ANOVAs, as appropriate. Tests were two-tailed unless a directional hypothesis was specified a priori; time-resolved comparisons were corrected using Benjamini–Hochberg FDR; and all effect sizes are reported as Cohen's d for t-tests. Where multiple related comparisons were conducted (e.g., across timepoints or feature spaces), p-values were corrected for multiple comparisons. In all figures, stars denote statistical significance at the following levels: (*$p < 0.05$; **$p < 0.01$; ***$p < 0.001$).

Table 1: Eyetracking data quality and exclusion metrics

	Excluded participants	Total included participants	Eyetracker confidence	Pretrial calibration accuracy	Valid trials per participant
Session 1	5	61	*M* = 0.75<br>*SD* = 0.08	*M* = 2.75 DVA<br>*SD* = 0.64	*M* = 52.8<br>(88%)<br>*SD* = 8.00
Session 2	3	26	*M* = 0.77<br>*SD* = 0.09	*M* = 2.86 DVA<br>*SD* = 0.57	*M* = 53.4<br>(89%)<br>*SD* = 6.08

Table 2: Demographic information for included participants.

	Total included participants	Age	Gender
Session 1	61	*M* = 20.67<br>*SD* = 3.42	36 Female<br>23 Male<br>2 Prefer NR
Session 2	26	*M* = 19.96<br>*SD* = 1.78	14 Female<br>12 Male

Supplementary Fig. 1a: Testing Session 1 Stimulus Set

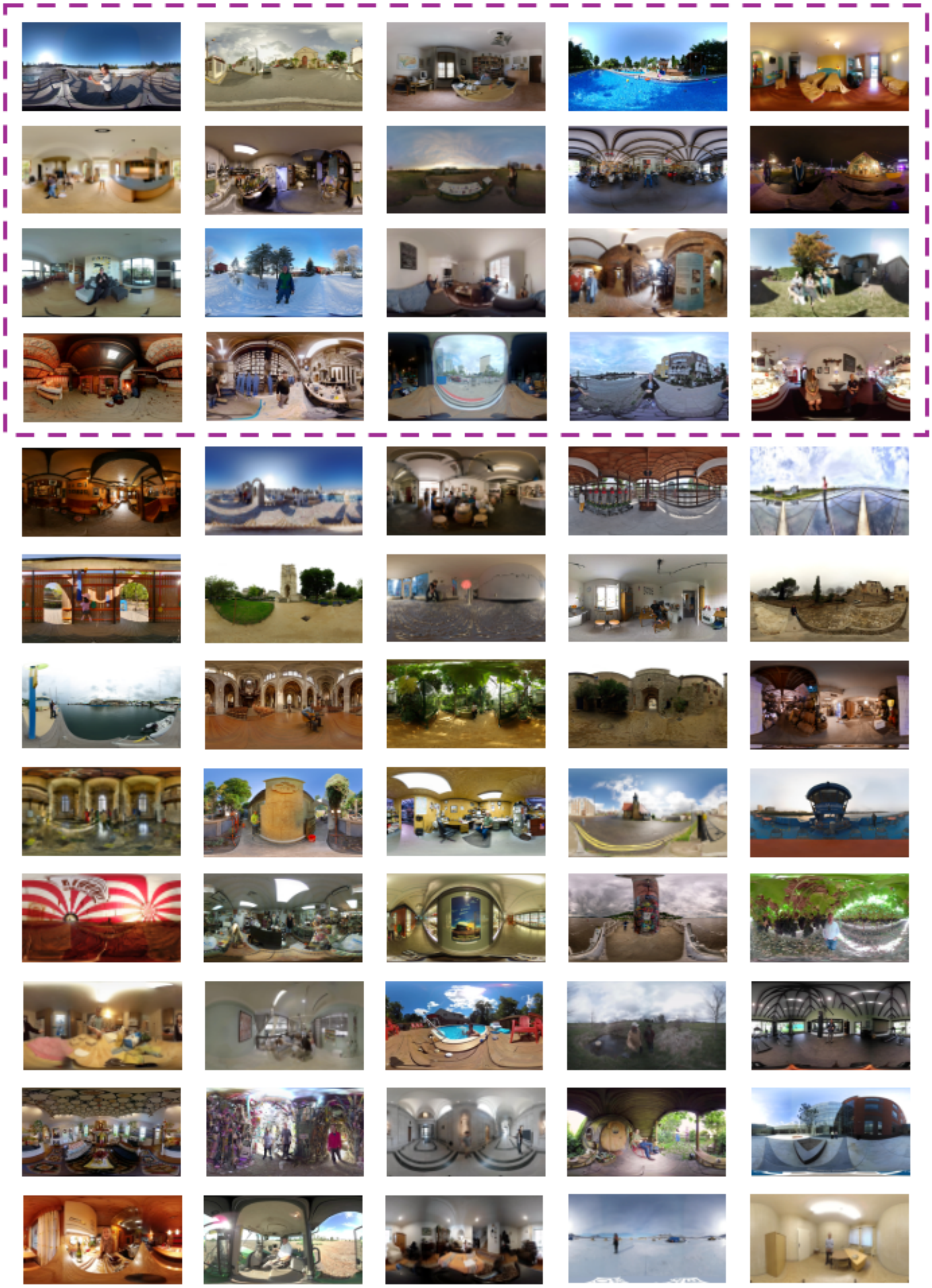

Supplementary Fig. 1b: Testing Session 2 Stimulus Set

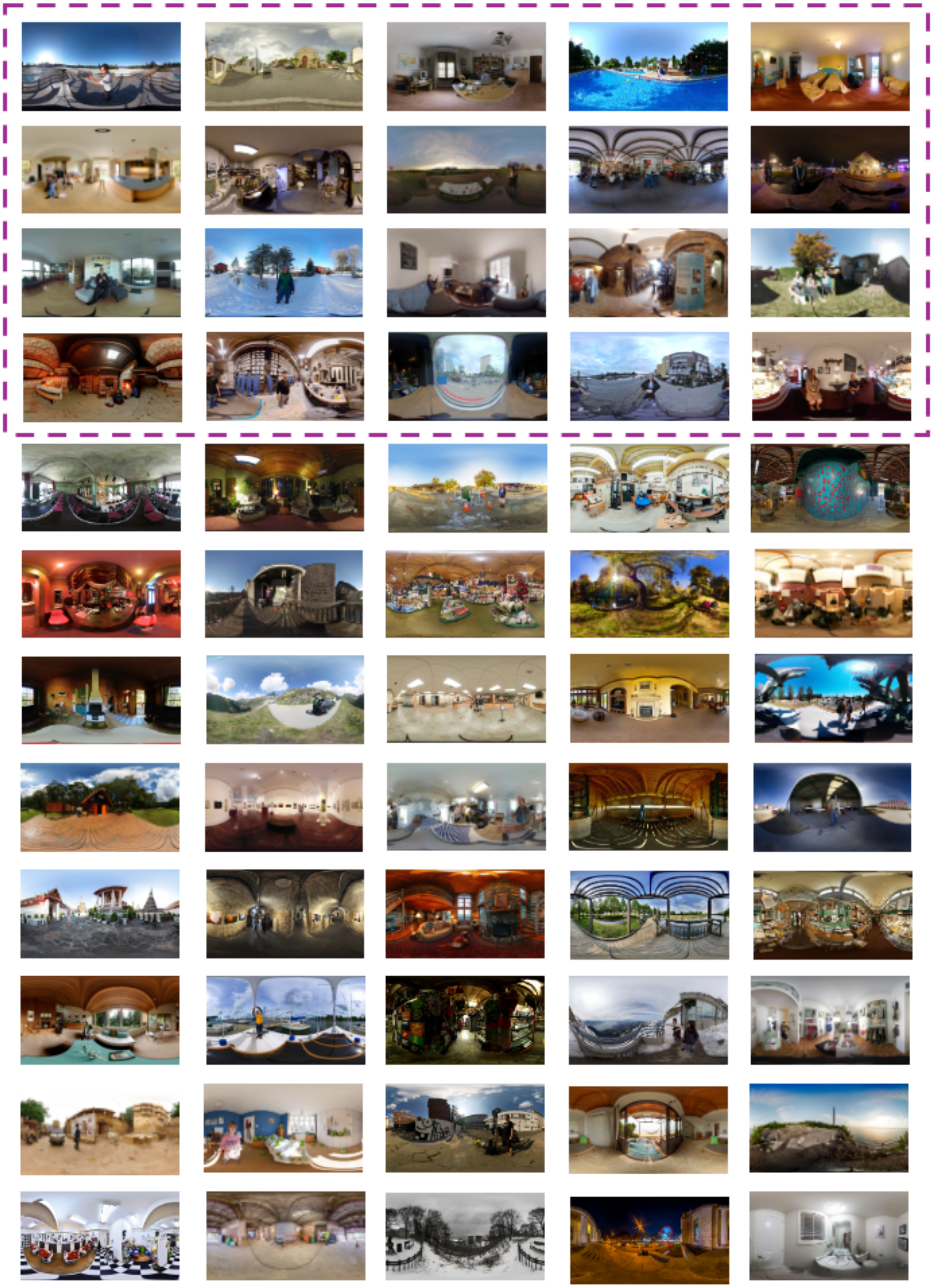

Supplementary Fig 1: Stimulus set. Participants actively explored immersive, 360° photospheres via headmounted virtual reality, displayed here in equirectangular format. **1a**, All participants who completed Session 1 (N = 61) viewed the same set of photospheres (N = 60), presented in randomized trial order. **1b**, All participants who returned for Session 2 (N = 26) viewed an additional, novel set of photospheres (N = 40), as well as 20 photospheres initially viewed in Session 1. Repeat photospheres, which were shown in both Session 1 and Session 2, are outlined in purple.

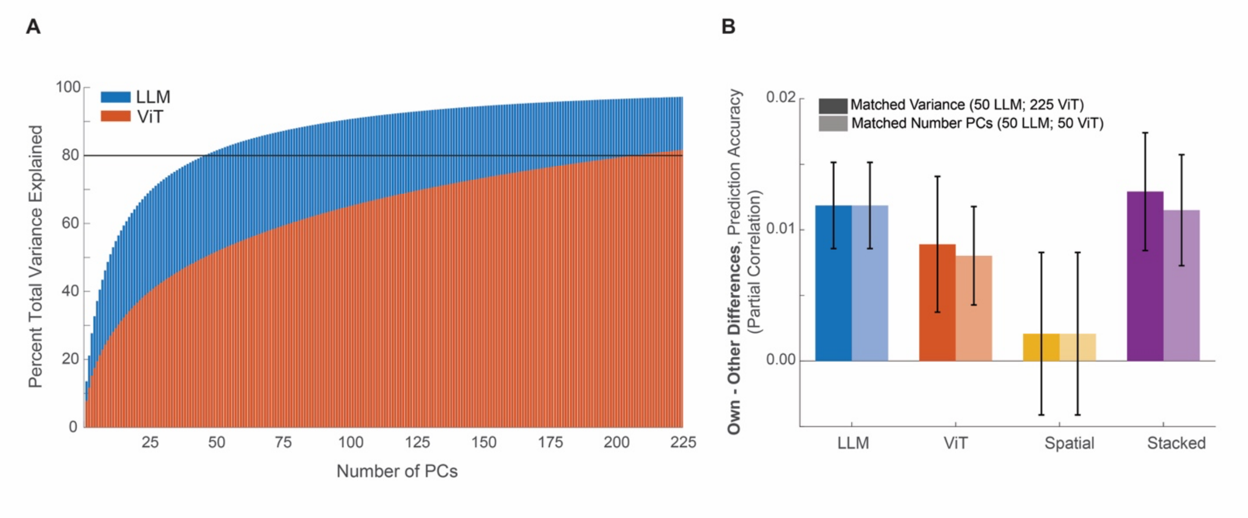

Supplementary Fig. 2: Conceptual feature contributions to individual gaze patterns are robust to principal component selection method. (A) Cumulative variance explained as a function of the number of principal components for LLM (BERT; blue) and ViT (orange) feature spaces. Due to differences in the intrinsic dimensionality of these feature spaces, 50 LLM PCs and 225 ViT PCs each capture approximately 80% of the variance in their respective embedding spaces. (B) Own-other differences in gaze prediction accuracy (measured as partial correlation) for spatial, visual (ViT), conceptual (LLM), and stacked feature spaces under two PC selection conditions: variance-matched (dark bars; 50 LLM PCs, 225 ViT PCs) and dimensionality-matched (light bars; 50 LLM PCs, 50 ViT PCs). Across all feature spaces, own-other prediction differences do not significantly differ between matching conditions, demonstrating that the contribution of conceptual features to observer-specific gaze behavior is not an artifact of PC selection criteria. Error bars represent 95% confidence intervals.

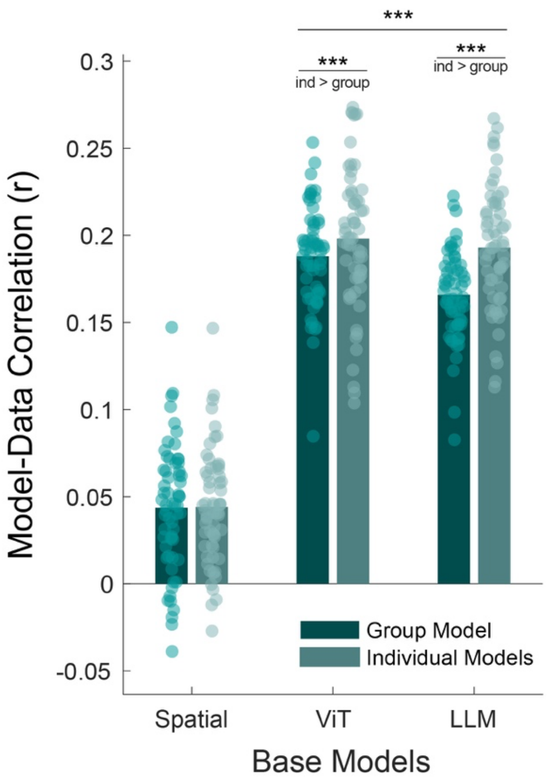

Supplemental Figure 3. Conceptual features show the largest individual-specific benefit across base models. For each base model (Spatial, Visual [ViT], Conceptual [LLM]), we compared prediction accuracy for models trained on group-averaged gaze data (Group Model, dark teal) versus models trained on individual participants' own gaze data (Individual Models, light teal). For Spatial base models, Group and Individual models performed comparably ($t(60) = -0.25$, $p > 0.8$, $d = 0.03$), indicating that spatial features primarily capture shared attentional structure. In contrast, Individual models significantly outperformed Group models for both Visual ($t(60) = 3.55$, $p < .001$, $d = 0.45$) and Conceptual features ($t(60) = 11.86$, $p < .001$, $d = 1.52$). The individual-specific benefit was significantly larger for Conceptual than Visual features ($t(60) = 7.01$, $p < .001$, $d = 0.90$), demonstrating that conceptual feature spaces preferentially capture variance specific to individual observers. Each dot represents one participant; bars show mean across participants. ***$p < .001$.
